# Efficacy of novel thermomechanically treated reciprocating systems for gutta-percha removal from root canals obturated with warm vertical compaction

**DOI:** 10.15171/joddd.2018.017

**Published:** 2018-06-20

**Authors:** cangul keskin, Evren Sarıyılmaz, Duygu Hazal Güler

**Affiliations:** ^1^Department of Endodontics, OndokuzMayıs University Faculty of Dentistry, Samsun, Turkey; ^2^Department of Endodontics, Ordu University Faculty of Dentistry, Ordu, Turkey

**Keywords:** Reciproc Blue, WaveOne Gold, Root canal filling material removal

## Abstract

***Background.*** The present study aimed to test the efficacy of novel reciprocating systems in terms of gutta-percha removal of roots obturated with warm vertical compaction technique.

***Methods.*** Ninety straight rooted maxillary incisors were enlarged with hand files up to a # 50/02 apical size and obturated using warm vertical compaction technique. The specimens were divided into four groups according to system used for filling removal, as Reciproc Blue, Reciproc, WaveOne Gold and hand-instrumentation. The residual filling materials and time required for root canal removal were calculated. Statistical analyses were performed using one-way analysis of variance and Tukey tests with 5% significance threshold.

***Results.*** There were no significant differences among Reciproc, Reciproc Blue and WaveOne Gold (P > 0.05). Hand-instrumentation group left significantly greater root canal filling material (P < 0.05). The time required for root canal filling removal was significantly shorter in the Reciproc group followed by WaveOne Gold, Reciproc Blue and hand-instrumentation groups (P< 0.05).

***Conclusion.*** Efficacy of Reciproc Blue, WaveOne Gold and Reciproc instruments for root canal filling removal were similar and superior to hand-instrumentation.

## Introduction

Nonsurgical retreatment aims to remove previous root canal filling material completely and regain access to the apical foramen with the goals of achieving proper disinfection by cleaning and re-shaping the root canal system.^1^ Complete removal of the previous root canal filling is essential for the success of retreatment and requires extensive time and effort.^2^ Various techniques and materials, including hand files, motor-driven instruments, chemical agents and heat, have been suggested for effective removal of root canal filling material.^3^ However, residual debris on root canal walls is regarded asa factor contributing to the outcome of the treatment.^2^

Several nickel-titanium (NiTi) motor-driven instruments have been suggested for removal of root canal filling materials to reduce operator fatigue and time.^4^ The use of instruments with a reciprocating motion has been reported to be a more rapid technique for the removal of root canal filling materials as compared to the use of a hand file or rotary instruments.^5^ Reciproc and Reciproc Blue (VDW, Munich, Germany)—instruments designed to function with a reciprocating movement—are produced from an M-wire and a novel thermally treated Blue wire, respectively. Apart from similar metallurgical compositions, both instruments have identical designs and kinematics. The manufacturer claims that both Reciproc and Reciproc Blue can be used for the removal of gutta-percha and carrier-based root canal fillings.^5^ WaveOne Gold (Dentsply Sirona, Ballaigues, Switzerland) is a novel reciprocating instrument manufactured using a gold wire that has replaced WaveOne file (Dentsply Sirona).^6^ Such metallurgical improvements have been reported to enhance the mechanical behavior of the instruments.^7,8^ However, to the best of our knowledge, no reports are available on the efficacy of the Wave One Gold and Reciproc Blue instruments in the removal of root canal filling materials during retreatment. The aims of this study were to compare the efficacies of Reciproc Blue, WaveOne Gold, Reciproc, and handinstrumentation techniques in the removal of filling materials from extracted human teeth by evaluating their root canal filling removal efficacy and time required for root canal filling removal. The null hypothesis was that there would be no significant differences between the tested instruments with respect to their efficacies in root canal filling removal and time required to reach the working length (WL).

## Methods

### Specimen selection and initial treatment

Clinical Research Ethics Committee of the local university approved the experimental protocol of the present study (ID number 2016/74). Ninety extracted human maxillary incisor teeth with a single patent canal, fully formed roots, and no calcification were used. The crowns were removed using a diamond disc (Brasseler, Lemgo, Germany) under watercooling to standardize the root lengths to 16 mm. Working length (WL) was determined by inserting a #15 K-file into the root canal until its tip was detected at the apical foramen under magnification via a loupe (×3.5) (Zeiss Eyemag Pro F, Oberkochen, Germany). The WLs were calculated by subtracting 1 mm from this measurement. The root canals were enlarged using manual instruments (DentsplySirona, Ballaigues, Switzerland) up to a #50/02 master apical file. Each time the instrument was changed, the root canals were flushed with 5 mL of 5.25% NaOCl (Wizard, Rehber Chem., Adana, Turkey). The final irrigation was achieved with 5 mL of 17% ethylenediaminetetraaceticacid (EDTA; H.P, ImidentMed, Konya, Turkey) for 1 minute, followed by 5 mL of distilled water, and 5 mL of 5.25% NaOCl. The root canals were dried with paper points and obturated using a warm vertical compaction technique (Elements Free, Kerr, Munich, Germany). A thin layer of sealer was spread on the root canal walls (AH Plus, Dentsply Sirona, Ballaigues, Germany) and a 50.02 gutta-percha cone (Aceone-Endo, Aceonedent Co., Geongg-Do, Korea) was inserted into the root canal confirming a tug back at the WL.Buchanan hand pluggers (Sybron Endo, Orange, CA, USA) were inserted into the root canal up to 3‒4 mm from the WL to remove thermoplasticized gutta-percha sequentially and compact the remaining gutta-percha vertically.The middle and coronal thirds of the root canals were obturated using BeeFill Backfill Unit. Root canal filling quality was checked by radiographs taken in both mesiodistal and buccolingual directions. The coronal surfaces were sealed with light-cured resin-modified glass-ionomer cement. The specimens were stored at 37°C and 100% relative humidity for 2 weeks to allow complete setting of the sealer.

### Retreatment procedures

The 10 teeth randomly assigned to the control group received no further treatment. The remaining 80 teeth were randomly divided into 4 groups according to the retreatment technique (n=20).

In the Reciproc Blue and Reciproc groups, the root fillings were removed with Reciproc Blue R25 and Reciproc R25 instruments, respectively. The instruments were attached to a VDW Silver endodontic motor (VDW Silver, Munich, Germany) and operated in the “Reciproc All” mode with a slow pecking motion until two-thirds of the root canal length was reached. Following 3 pecking motions with 3 mm amplitude applied with light pressure, the instrument was pulled out of the canal to clean the debris accumulated in the flues. This cycle was repeated until the WL was reached. Then, the final preparations were completed with Reciproc Blue R40 and R50 in the Reciproc Blue group and Reciproc R40 and R50 in the Reciproc groups.

In the Wave One Gold Primary group, the instrument was operated with a VDW Silver motor in a slow in-and-out pecking motion, similar to the one described in the Reciproc groups. Final preparation was carried out with Wave One Gold Medium and Large instruments, respectively.

In the handinstrumentation group, the coronal root canal obturatingmaterial was removed using#3 Gates-Glidden drills;#35, #30 and #25 Hedstrom files (H-file) (Dentsply Sirona) were used in circumferential, quarter-turn push-pull and filing manners, respectively, to remove the obturatingmaterials until the WL was reached. After the WL was reached the root canals were prepared with circumferential filing using #30, #35, #40, #45 and #50 H-files.

The removal of the obturatingmateriakwas considered complete when no further material was observable on the flutes of the instrument, which was checked using a loupe (Carl Zeiss) at ×3.5 magnification. Irrigation was performed on each specimen with 20 mL of 2.5% NaOCl solution. A single operator performed all the root canal treatment and retreatment procedures. No solvent was used during the experiments. The instruments were used only once for each specimen and then discarded. The final irrigation was achieved using 5 mL of 17% EDTA for 3 minutes, followed by 5 mL of 2.5% NaOCl. The time for each procedure, starting from the moment the instruments were introduced into the canal until no residual filling material could be observed, was recorded using a chronometer (excluding the time required for irrigation and instrument changes).

## Evaluation of root canal filling removal

**Figure 1:**
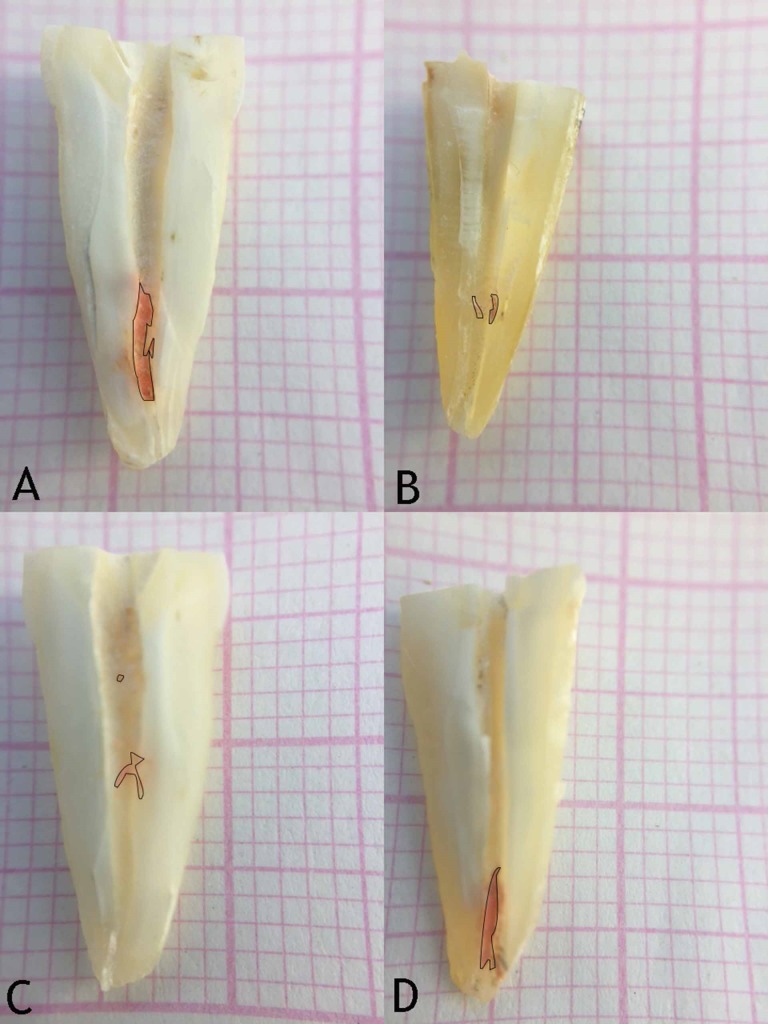
Representative images of the remaining root canal filling materials in the experimental groups; (A) Hand instrumentation (B) Reciproc (C) Reciproc Blue (D) WaveOne Gold

Longitudinal grooves were prepared on the buccal and lingual surfaces of the specimens. The two halves were split using a spatula and each root half was visualized at ×10 magnification under a stereomicroscope (Nikon, SMZ 745T, Tokyo, Japan). The images were analyzed using Image J software (National Institutes of Health, Bethesda, MD, USA), whereby the percentage of the remaining root canal filling material was calculated (Figure 1).

### Statistical analysis

The mean percentage area of the residual obturating material and the time required for removalof the obturating material were analyzedwith one-way ANOVA and Tukey tests after the Shapiro-Wilk test revealed that both sets of data displayed normal distributions. The level of significance was set at 5% for all the statistical tests, which were performed using SPSS 21.0 (SPSS Inc., Chicago, IL, USA).

## Results

An examination of the specimens in the control group indicated that the separation procedure did not lead to a dislodgement of the root canal filling material. All the specimens contained residual filling material (Table 1). The handinstrumentation group contained a significantly greater amount of root canal filling material compared to the Reciproc Blue, Reciproc, and WaveOne Gold groups (P<0.05). There were no significant differences between the Reciproc, ReciprocBlue and WaveOne Gold groups (P>0.05).

**Table 1:** Mean and standard deviations of residual filling material (% area) and time(s) required for retreatment (s) of the experimental groups.

**Groups**	**Mean % (standard deviation)**	**Time**	***n***
Reciproc	2.860 (0.91)^a^	216.90 (24.22)^a^	20
Reciproc Blue	2.919 (1.42)^a^	278.23 (12.20)^b^	20
WaveOne Gold	3.070 (1.44)^a^	271.36 (18.90)^b^	20
Hand instrumentation	8.710 (1.99)^b^	365.70 (21.60)^c^	20

Different superscript letters indicate statistically significant difference (P<0.05).

Time required for root canal filling removal was significantly shorter in the Reciproc group followed by the WaveOne Gold, Reciproc Blue and handinstrumentation groups (P<0.05). No significant difference in time required could be detected between the Reciproc Blue and WaveOne Gold groups (P>0.05). The handinstrumentation group required the longest time for filling removal (P<0.05).

No instrument fracture occurred during the removal of root canal fillings in the groups.

## Discussion

Thus far, root canal filling remnants have not been directly associated with retreatment failure; however, it is logical and reasonable to remove the maximum amount of filling material possible to promote disinfection and cleaning. Obturating material residues might harbor microorganisms, which are responsible for the failure of the initial treatment and persistent endodontic infections.^9,10^ The efficacies of several techniques and materials have been evaluated for root canal filling removal; none of them could eliminate the root canal filling remnants completely.^5,11,12^ The present study compared the efficacies of novel reciprocating instrument and handinstrumentation techniques for the removal of root canal fillings as well as the time required for removal of obturating materials. Significant differences were found between the experimental groups with respect to their root canal removal efficacy and time required to reach the WL; therefore, the null hypothesis was rejected.

In the present study, the specimens were sectioned longitudinally into two separate halves and examined under a stereomicroscope. The cleaving technique has been reported to be superior to the 2D radiographic analysis. However, the possibility of root canal filling dislodgement during the sectioning procedure is a disadvantage.^13^ In the present study, an examination of the control group verified that none of the root canal fillings was dislodged during sectioning.

The use of reciprocating instruments with #25 apical diameter is the first technique that has been suggested for the removal of root canal fillings up to the WL.^14,15^ In the present study, the efficacies of several novel reciprocating instruments in removing root canal fillings up to the WL were compared; the experimental design was similar to that used in several previous studies.^11,16^ The results of the present study revealed that none of the tested instruments removed the root canal fillings completely, which is consistent with previous reports.^5,11^ This implies that re-instrumentation of the root canal system to a larger diameter or more conservatively the use of additional and more effective irrigation protocolsis required to remove residual filling materials .^17,18^

Reciproc Blue and Reciproc have identical designs and operational modes; the only difference between the two instruments is in the manufacturing process. In the case of Reciproc Blue, the thermomechanical manufacturing process used results in the formation of a proprietary-specific oxide surface layer that gives the instrument its blue color and enhances its mechanical properties, such as cyclic fatigue resistance and flexibility.^7,8^ The results of the present study showed that Reciproc Blue and Reciproc exhibited similar cleaning efficacy during root canal filling removal. This observation supports the claim of the manufacturer that Reciproc Blue can also be used for retreatment.^19^ However, Reciproc Blue took significantly longer to reach the WL than Reciproc did. This might be attributed to the higher flexibility and ductility of the instrument as compared to the M-wire-based Reciproc.^7^ The difference between the mean operating times of the two instruments was less than a minute, which might not be a significant difference from a clinical point of view. Since the cyclic fatigue resistance of Reciproc Blue is higher than that of Reciproc, it might be preferable to use Reciproc Blue in the retreatment of curved root canals.^7^

In the present study, it was observed that the handinstrumentation technique left significant amounts of root canal filling remnants and took the longest time to reach the WL, as compared to the reciprocating instruments. These results are consistent with the observations of Helvacioglu-Yigit.^12^ On the other hand, in a recent systematic review, the efficacy of NiTi motor-driven instruments was found to be similar to that of hand-instrumentation techniques.^20^ The differences between the results of these studies might be attributed to the anatomical differences between the roots, different root canal filling materials and techniques, and different removal techniques.

The cleaning efficacy of a NiTi instrument is associated with its design properties such as cutting efficacy, taper and cross-sectional shape.^21,22^ A smaller cross-sectional shape and less taper creates more space between the instrument and the dentin, provides efficient displacement of the debris/root canal filling residues from the apical direction to the coronal direction, and improves the cutting ability.^23^ In the present study, no significant differences could be detected between the Reciproc, Reciproc Blue, and WaveOne Gold groups. Reciproc Blue and Reciproc have S-shaped cross-sections, whereas WaveOne Gold has a parallelogram-shaped cross-section. The parallelogram-shaped cross-section has been reported to provide efficient space for improved cutting, loading and transportation of debris in the coronal direction.^24^ Moreover, WaveOne Gold instruments exhibit less taper than Reciproc Blue and Reciproc instruments. The offset cross-sectional shape and reduced taper might account for the good root canal removal efficacy of WaveOne Gold by favoring penetration into and coronal extrusion of the filling material.

Stereomicroscopic examination of the root halves is a destructive technique that produces two-dimensional images, which might present limitations to the present study. Micro-computed tomography emerged as a valuable non-destructive imaging technique, which allows quantitative evaluation of residual root canal fillings and provides comparative data before and after root canal retreatment.^20^ However, limitations of micro-computed tomography includes accessibility, high cost and the necessity of high technical knowledge.

## Conclusions

Within the limitations of this in vitro study, all the instrument systems tested left root canal filling remnants on the root canal walls. The efficacies of Reciproc Blue, Reciproc, and WaveOne Gold instruments for root canal filling removal were found to be similar and superior to that of handinstrumentation.

## Acknowledgments

None.

## Authors’ contributions

CK, ES, and DHG contributed to the concept and the design of the study. CK and DHG performed the experiments. ES contributed to the interpretation of the data. CK drafted the manuscript. All authors contributed to the critical revision of the manuscript, and have read and approved the final paper.

## Funding

Not applicable.

## Competing interests

The authors declare no competing interests with regards to the authorship and/or publication of this article.

## Ethics approval

The study protocol was approved by local university clinical researches ethical board with ID number 2016/74.
